# Development of an Innovative 3D Cell Culture System to Study Tumour - Stroma Interactions in Non-Small Cell Lung Cancer Cells

**DOI:** 10.1371/journal.pone.0092511

**Published:** 2014-03-24

**Authors:** Arno Amann, Marit Zwierzina, Gabriele Gamerith, Mario Bitsche, Julia M. Huber, Georg F. Vogel, Michael Blumer, Stefan Koeck, Elisabeth J. Pechriggl, Jens M. Kelm, Wolfgang Hilbe, Heinz Zwierzina

**Affiliations:** 1 Department of Internal Medicine V, Medical University Innsbruck, Innsbruck, Tyrol, Austria; 2 Department of Anatomy, Histology and Embryology, Medical University Innsbruck, Innsbruck, Tyrol, Austria; 3 InSphero AG, Schlieren, Canton of Zürich, Switzerland; University of Bergen, Norway

## Abstract

**Introduction:**

We describe a novel 3D co-culture model using non-small cell lung cancer (NSCLC) cell lines in combination with lung fibroblasts. This model allows the investigation of tumour-stroma interactions and addresses the importance of having a more in vivo like cell culture model.

**Methods:**

Automation-compatible multi-well hanging drop microtiter plates were used for the production of 3D mono- and co-cultures. In these hanging drops the two NSCLC cell lines A549 and Colo699 were cultivated either alone or co-cultured with lung fibroblasts. The viability of tumour spheroids was confirmed after five and ten days by using Annexin V/Propidium Iodide staining for flow-cytometry. Tumour fibroblast spheroid formation was characterized by scanning electron microscope (SEM), semi-thin sections, fluorescence microscope and immunohistochemistry (IHC). In addition to conventional histology, protein expression of E-Cadherin, vimentin, Ki67, fibronectin, cytokeratin 7 and α-smooth muscle actin (α-SMA) was investigated by IHC.

**Results:**

Lower viability was observed in A549 monocultures compared to co-cultures, whereas Colo699 monocultures showed better viability compared to co-cultures. Ki67 expression varied significantly between mono- and co-cultures in both tumour cell lines. An increase of vimentin and decreased E-Cadherin expression could be detected during the course of the cultivation suggesting a transition to a more mesenchymal phenotype. Furthermore, the fibroblast cell line showed an expression of α-SMA only in co-culture with the cancer cell line A549, thereby indicating a mesenchymal to mesenchymal shift to an even more myofibroblast phenotype.

**Conclusion:**

We demonstrate that our method is a promising tool for the generation of tumour spheroid co-cultures. Furthermore, these spheroids allow the investigation of tumour-stroma interactions and a better reflection of in vivo conditions of cancer cells in their microenvironment. Our method holds potential to contribute to the development of anti-cancer agents and support the search for biomarkers.

## Introduction

Due to the increasing understanding of the mechanisms relevant to the genesis of cancer, we are experiencing a transition from disease to target-oriented therapy. As a consequence, the future of molecular targeted therapy of cancer lies in identifying subsets of patients who benefit from particular therapies that hit specific structures expressed by the malignant cell. One major hurdle for the development of these individualized therapeutic regimens, however, is the limited availability of predictive in vitro models. The critical challenge is to develop cell culture models better reflecting in vivo conditions and thereby supporting the investigation of predictive biomarkers that have the potential of enhancing the value of cancer medicines and reducing the size, cost and failure rates of clinical trials.

Non-small cell lung cancer (NSCLC) is one of the leading causes of cancer deaths in male and female patients worldwide.

Only 15%–20% of them are diagnosed at an early stage [1]. The prognosis remains poor with a 5-year survival rate ranging from approximately 60% for stage I to less than 5% for stage IV tumours [2].

Patients diagnosed with locally advanced disease require multimodality treatment to achieve long-term remission or even cure while patients with metastatic disease receive platinum-based chemotherapy either alone or in combination with EGFR or alk inhibitors [3]–[5].

Numerous other molecular targeted agents have been tested in clinical trials but failed to show a benefit for patients regarding progression free survival and overall survival [6]. Several of these trials aimed to define biomarkers in a prospective or retrospective way but only a very limited number have been identified [7], [8].

So far cell-based assays to explore cell biology and drug efficacy aimed at growing cells on two-dimensional plastic surfaces or in single cell suspension [4]. The biology of cells, however, being profoundly influenced by their micro-environment require cell based assays that reflect the effects of factors such as the extracellular matrix (ECM), cell-cell contacts, cell-matrix interactions, cell polarity and oxygen profiles [5]–[8].

Conventional two dimensional (2D) cell culture systems grown on artificial plastic surfaces have major limitations. For example they require high non-physiological fetal calf serum (FCS) concentrations and refeeding by changing medium every 2-3 days. In contrast to that, 3D techniques avoid plastic surfaces allowing cells to form their ECM and require significantly reduced FCS concentrations. Not only cell morphology but also drug sensitivity of cancer cells in 2D systems is different compared to in 3D cell cultures [9], [15]. Cells cultivated on plastic surfaces usually exhibit an increased sensitivity to cytotoxic drugs, while compounds targeting cell - cell adhesions, cell maturation, epithelial-mesenchymal transition (EMT) and stemness features often show a decreased efficacy in 3D cell culture. Thus 3D cell culture models reflect in vivo tumour growth more reliably and may provide better read outs for drug testing [9], [15], [10].

Most 3D systems use cell spheroid aggregates and scaffold culture systems. These systems support 3D cell growth by artificially produced extracellular homologues (e.g. collagen, matrigel, scaffolds) facilitating cell adhesion and aggregation. Other 3D systems use liquid overlay technologies, fibre meshwork made of biocompatible polymers, solid or porous beads or extracellular matrices and their substitutes and require the addition of artificially produced supplements for achieving 3D growing cell cultures [16]–[19].

The hanging drop technique is a well-established cell culture method to form spherical microtissues from immortalized and primary cell lines [20]–[22]. In contrast to most liquid overlay technologies, the hanging drop method allows the precise control over the initial cell composition in each microtissue [23], [24]. To generate multi-cell type co-culture microtissues neither additional supplements nor artificial scaffolds mimicking extracellular matrix components (e.g. collagen matrigel) are required.

Based on an automation and high-throughput compatible hanging drop technology we established an organotypic co-culture models composed of two different non-small cell lung cancer (NSCLC) cell lines in combination with lung fibroblasts. This method enables not only the investigation of tumour-stroma interactions and represents a more in-vivo like 3D cell culture model to study drug tumour interactions but can also be implemented in future drug discovery campaigns.

## Materials and Methods

### Cell culture

The NSCLC cell lines A549 and Colo699 (DSMZ, ACC107, ACC196) and the lung fibroblast cell line SV-80 (CLS, 300345) were used for our experiments. For 2D culture, cell lines were cultured as monolayer in DMEM low glucose (PAA, Pasching, Austria) supplemented with 10% FCS (Sigma-Aldrich, Munich, Germany, Lot 010M3396) and 100 U/ml penicillin,100 μg/ml streptomycin solution, 2 mM L-Glutamine (PAA). Cells were cultivated at 37°C in a humidified 5% CO_2_-containing atmosphere.

### 3D cell culture

For the production of 3D mono- and co-cultures GravityPLUS™ microtissue culture system (InSphero AG, Zürich, Switzerland) were used. The plate consists of a frame with 8×12 well inserts and a peripheral channel filled with 2–3 ml sterile water to provide a humidity barrier that reduces evaporation of medium from the drops. This frame is placed on a bottom-tray which is filled with 5 ml 0.2× phosphate buffered saline (PBS) (PAA) with 0.1% Triton X-100 (Sigma), again to reduce evaporation from the droplets. The well is divided in three elements, (i) the inlet on which the pipette tips are placed directly on the surface of the well, (ii) the culture compartment and (iii) a microchannel connecting the inlet and the culture compartment. Each well is spring-loaded to compensate for any slight variations in tip heights of 96-well pipette heads.

After cells had been grown subconfluently in cell culture plates (Falcon) they were seeded into the hanging drops. Then, cells were washed once with PBS and digested with 1× Accutase (PAA) for ten minutes at 37°C. Thereafter, the digestion was stopped and cells washed once with 20 ml of cell culture medium. Then, cells were counted and seeded in 40 μl drops in various cell counts (Monocultures: SV-80 and A549: 2500 cells/40 μl; Colo699: 1250 cells/40 μl; co-cultures: carcinoma cell: fibroblast ratio 1∶2/40 μl). Medium exchange was performed every four days and the spheroids were harvested after five and ten days by loading 100 μl of PBS (PAA) onto the microcapillaries to flush out the spheroids. The falling drops containing the spheroids were then collected with a 96-well U-bottom plate (Falcon) that is placed underneath the microcapillary plate.

#### CFSE staining of fibroblasts

For the fluorescent labeling of fibroblasts the CellTrace™ CFSE Cell Proliferation Kit (Molecular Probes, Invitrogen) was used. Fibroblasts were dissolved and adjusted to 1×106 in 1 ml of PBS/0,1% bovine serum albumin (BSA).CFSE solution was added to achieve a final working concentration of 10 μM. Cells were then incubated for 10 minutes at 37°C. Afterwards, the staining was quenched with five volumes of ice cold cell culture medium and incubated for 5 minutes on ice. Then cells were washed three times with cell culture medium. Labeled Fibroblasts were now seeded as co-cultures with carcinoma cells as described in the 3D cell culture section.

### Flow-cytometry

Eight microtissues, harvested in 96-well plates were transferred into 5 ml FACS tubes (Falcon) and washed once with 2 ml PBS. Afterwards, microtissues were solubilized into single cell suspension by adding 300 μl Accumax (Miltenyi Biotech) per microtissue incubating them for 20 minutes at 37°C. After ten minutes microtissues were mixed and pipetted up and down to obtain a homogen solution. This procedure was repeated after 20 minutes of incubation. Now the digestion was stopped with 2 ml complete cell culture medium, then the cells were centrifuged at 300 g for ten minutes and washed twice with 0,5% BSA/PBS. Afterwards, cells were stained with 4 μl Propidium Iodide (PI) staining solution and 4 μl of Annexin V APC. Staining was performed in 100 μl Annexin Binding Buffer taken from the Annexin V Apoptosis Detection Kit I (Becton Dickinson) for 15 minutes. Finally, cells were analyzed on a BD FACS Calibur immediately. Each data measurement was made up from eight pooled microtissues, repeating the whole procedure independently three times.

### Volume calculation of microtissues

Six microtissues of mono- and co-cultures were evaluated by an inverted light microscope (Motic AE31) from day one to ten. After five, seven and ten days, two diameters (d) of each microtissue were measured and the volume was calculated by the formula V = 4/3*Π*r^3^, where r = ½*√ d1*d2 [25]. Volume measurement was repeated three times. Thereafter, mean and standard deviation was calculated and statistical significance tested with the student's t-test in Microsoft excel.

### Immunohistochemical analyses

#### Sample preparation

Microtissues were rinsed in PBS and then immediately fixed by immersion in cold 4% paraformaldehyde (PFA) in PBS, for four hours at room temperature. Microtissues were then rinsed in PBS again and embedded in 2% agarose (Invitrogen). Excess agarose was removed with a scalpel, and the agarose blocks containing the microtissues were dehydrated in graded alcohols and embedded in paraffin wax (Paraplast regular, Sigma-Aldrich, St. Louis, MO, USA)A Microm ERGO Star Rotary microtome (Microm, Walldorf, Germany) was used to take serial sections of 4 μm thickness. A three to four series of paraffinized sections were then mounted in a meandering pattern on SuperFrost Plus slides (Menzel-Gläser, Braunschweig, Germany) and dried overnight, then baked at 60°C for one hour to adhere the sections firmly to the slides. For cyto-architectural orientation, every tenth slide was hematoxylin/eosin stained (HE) using a Shandon Varistain 24-4 Slide Stainer (Histocom Vienna, Austria).

#### Antisera

Hosts, dilutions, incubation times and sources of primary antibodies as well as heat-induced epitope retrieval (HIER) are listed in Table 1.

**Table 1:** Primary antibodies used for immunohistochemistry.

Antibody (clone)	Host	Dilution	Antigen unmasking	Incubation time	Source (# number)
**E-Cadherin (36B5)**	mouse mAB	ready-to-use	CC1 standard	60 min	Novocastra (E601)
**Vimentin (V9)**	mouse mAB	ready-to-use	CC1 short	60 min	Linaris (E034)
**α-SMA (1A4)**	mouse mAB	ready-to-use	none	12 min	Linaris (E046)
**Ki-67 (30-9)**	rabbit mAB	ready-to-use	CC1 standard	60 min	Ventana (790-4286)
**Fibronectin (IST-9)**	mouse pAB	ready-to-use	CC1 standard	60 min	Abcam (ab6328)
**Cytokeratin 7(SP52)**	rabbit mAB	ready-to-use	CC1 standard	60 min	Ventana (790-4462)

### Immunocytochemistry

Immunocytochemistry was carried out on 4 μm sections of paraffin-embedded microtissues in a Ventana Roche Discovery Immunostainer (Mannheim, Germany) according to the DAB-MAP discovery research standard procedure. If required, antigen retrieval was initiated by heat-induced unmasking of the epitopes while the slides were immersed in accordance with the manufacturer's instructions (short or standard for different incubation times) in EDTA buffer (Cell Conditioning Solution CC1, Ventana). After incubation of the sections with the primary antibodies (listed in Table 1.) at 37°C, a biotinylated immunoglobulin cocktail of goat anti-mouse IgG, goat anti-mouse IgM, goat anti-rabbit IgG and protein block (Discovery Universal Antibody, Ventana) was applied for 30 minutes at room temperature. The detection was achieved using the DAB-MAP Detection Kit (Ventana) according to the diaminobenzidine (DAB) development method. Sections were finally counterstained with hematoxylin (Ventana) for four minutes. Subsequently, sections were manually dehydrated in downgraded alcohol series, cleared in xylene and cover slipped permanently with Entellan (Merck, Darmstadt, Germany).

Digital images of HE- and immunostained slides were acquired in AxioVision microscope software linked to an AxioCam HRc color camera and an AxioPlan 2 microscope (Zeiss, Jena, Germany).

Ki67 positivity was determined by counting Ki67 positive and negative cell nuclei in three different spheroids of A540 and Colo699 in mono- and co-cultures representatively. Thereafter, the percentage of positive cell nuclei to whole cell number was calculated. Mean and standard deviation and statistical significance was calculated as described before.

### Fluorescence microscope

Microtissues in mono- and co-cultures were harvested as described above and washed once with PBS. Afterwards, tumour microtissues were fixed with 4% PFA for two hours at 4°C and washed three times with PBS. Now, they were blocked by incubation with PBS containing 2% BSA (Sigma) /0,3% Triton X-100 (Roche) for one hour at room temperature. Microtissues were then incubated 40 minutes with 50 μg/ml phalloidin Atto 633 (Sigma) at room temperature in the dark. Thereafter, they were washed again five times with PBS and were then incubated five minutes with 1,5 μg/ml DAPI (Invitrogen) at room temperature in the dark. After washing one time with PBS microtissues were brought onto an object slide with fluorescent mounting medium (Dako Glostrup, Denmark) and were analysed on a LSM 510 Meta Confocal Microscope (Zeiss).

### Scanning Electron Microscopy (SEM)

Tumour microtissues were fixed with 2.5% glutaraldehyde (BioChemika Fluka) in 0.1 M phosphate buffer (pH 7.4). After a brief wash in PBS, followed by post-fixation for one hour with 1% aqueous osmium tetroxide (ReagentPlus; Sigma-Aldrich), samples were gradually dehydrated with ethanol. After drying (CPD 030, Bal-Tec), microtissues were mounted on aluminium stubs with double-sided adhesive tape, sputter-coated with 10-nm gold/palladium (Au / Pd) (Bal-Tec) and examined with a field emission scanning electron microscope (Gemini 982; Zeiss, Goettingen, Germany).

### Semi-thin sections

Tumour microtissues were processed according to standard technique for transmission electron microscopy. Microtissues were fixed in Karnovsky's fixative (2.5% glutaraldehyde and 2% paraformaldehyde in 0.1 M cacodylate buffer) overnight and then washed three times (10 minutes each) in 0.1 M cacodylate buffer, followed by fixation with 1% osmium tetraoxide in 0.05 M cacodylate buffer at 4°C for 1 hour. Excessive osmium tetraoxide was removed by rinsing the microtissues three times (10 minutes each) in 0.1 M cacodylate buffer. Microtissues were then dehydrated with ethanol (2×70% ethanol each 30 minutes, 2×96% ethanol each 30 minutes, 2×100% ethanol each 30 minutes) and acetone (2× each 30 minutes) prior to incubation in a dilution of liquid epoxy resin (Epon medium mixture: 20 ml EMbed-812, 16 ml DDSA, 8 ml NMA and 1.2 ml BDMA; Electron Microscopy Sciences, Munich, Germany) and acetone in a ratio of 30% to 70% for three hours. The microtissues were infiltrated with a mixture of Epon and acetone in equal shares at 4°C overnight in closed vials. The next day, the liquid was replaced by a mixture of 70% epoxy resin and 30% acetone for three hours. Subsequently, microtissues were incubated twice in 100% Epon (3 hours and at 4°C overnight). Then, pure epoxy resin was changed, and the microtissues were infiltrated with Epon in a vacuum chamber for 2 hours. Microtissues were then transferred to an embedding mold, labeled, and placed in a vacuum chamber for an additional 1 hour. Microtissues were transferred to an incubation chamber at 60°C for 48 hours to increase the polymerization of the epoxy resin. One-micrometer sections were cut on a Leica Ultracut microtome, stained with toluidine blue at 60°C, and then examined using a light microscope.

## Results

### Tumour microtissue formation

**Figure 1:**
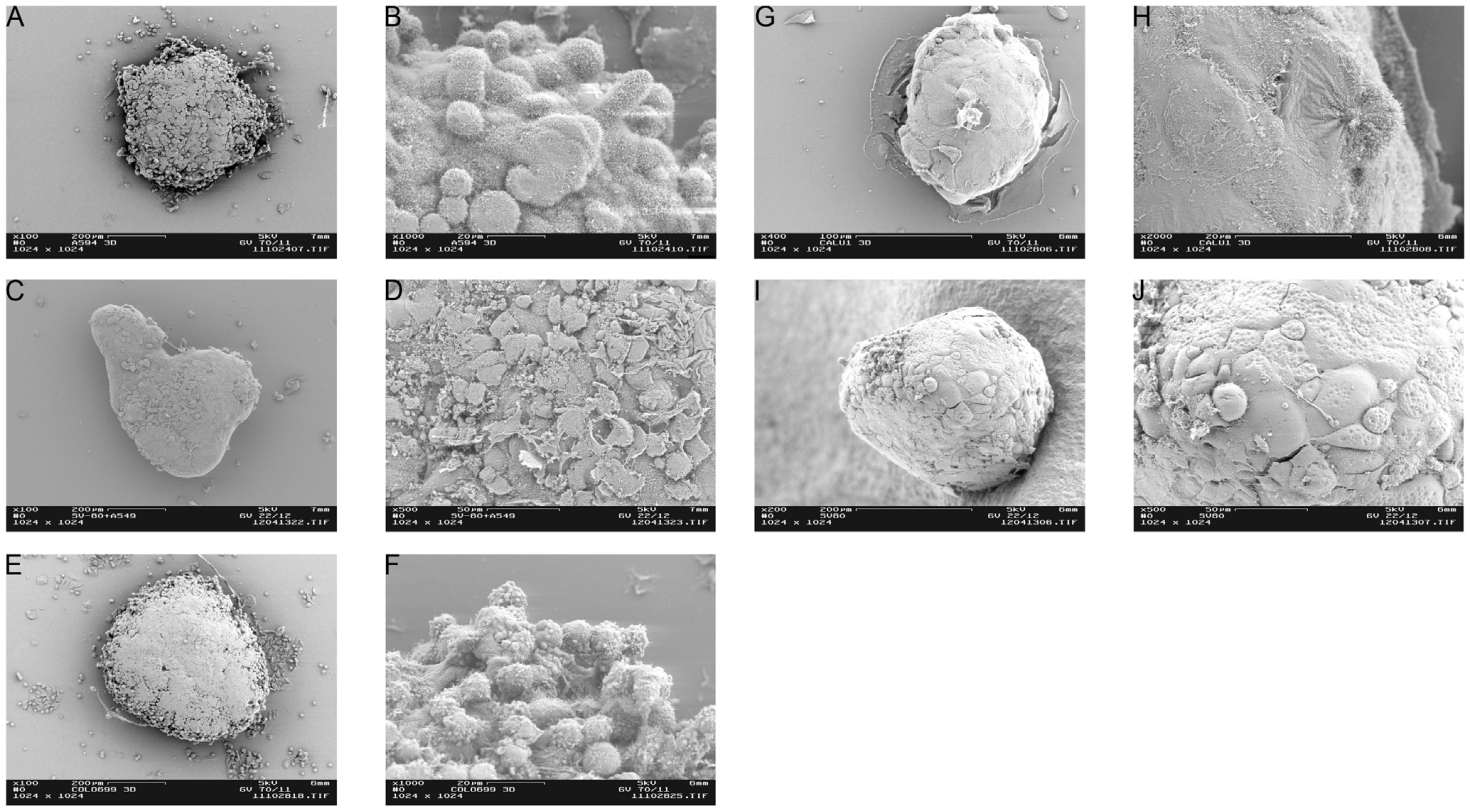
Tumour microtissue formation. SEM pictures were taken after ten days: Monocultures of A549 and SV80 were seeded in a ratio of 2500 cells/40 μl, whereas Colo699 monocultures were cultured in form of 1250 cells/40 μl. All co-cultures were seeded in a carcinoma cell: fibroblast ratio of 1∶2/40 μl (A549 co-cultures: 2500 cancer cells +5000 fibroblasts / Colo699 co-cultures: 1250 cancer cells +2500 fibroblasts). (A/B) A549 monocultures, (C/D) A549 co-cultures; (E/F) Colo699 monocultures, (G/H) Colo699 co-cultures; (I/J) SV80 monocultures. All co-cultures displayed a more homogenous and rounder microtissue surface compared to monocultures.

**Figure 2:**
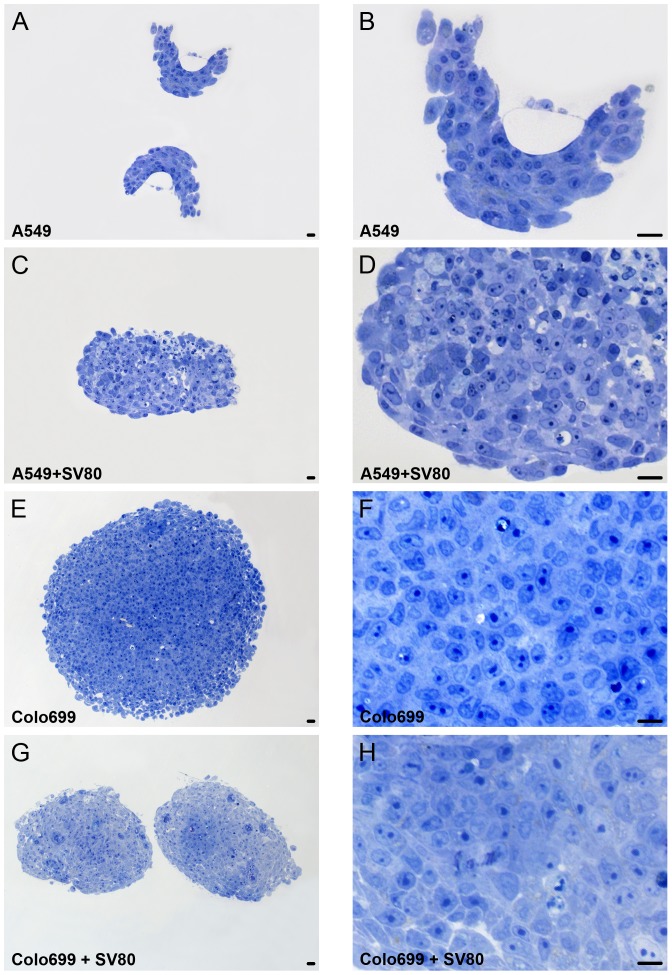
Tumour microtissue architecture. Semi-thin sections (1 μm slices) of A549/Colo699 mono- and co-cultures after ten days of cultivation stained with toluidine. Bar in all pictures: 20 μm. A549 cells form rugged spheroids resembling a more multi-layered tissue and exhibit a rough surface. Colo699 monocultures reveal a spheroidal structure with a loose surface. When fibroblasts are added both cell lines build a more compact microtissue with a regular surface.

At first, we investigated the arrangement of tumour cells and fibroblasts into spheroids during the culture period. For this purpose SEM pictures (figure 1) of A549 and Colo699 mono- and co-cultures were taken after ten days of cultivation. In addition, A549/Colo699 mono- and co-cultures were prepared for semi-thin sections and stained with toluidine blue (figure 2).

As shown in SEM pictures and semithin sections, A549 cells form rugged spheroids resembling a more multi-layered tissue and exhibit a rough surface. After five days microtissues had not fully formed and got easily disrupted during harvesting procedure. In contrast, when used in co-culture these cells form tight spheroids with smooth surfaces. In semithin sections fibroblasts may be detected as cells that have a flat morphology mainly arranged near the surface of the microtissues.

During the course of cultivation Colo699 monocultures reveal a spheroidal structure with a loose surface. When fibroblasts are added these spheroids build a more compact spheroid with a regular surface. As all cells had the same morphology, however, tumour cells and fibroblasts could not be discriminated in semithin sections.

In contrast to the cancer cell lines, SV80 fibroblasts form small tight looking spheroids with a smooth surface when cultivated alone.

**Figure 3:**
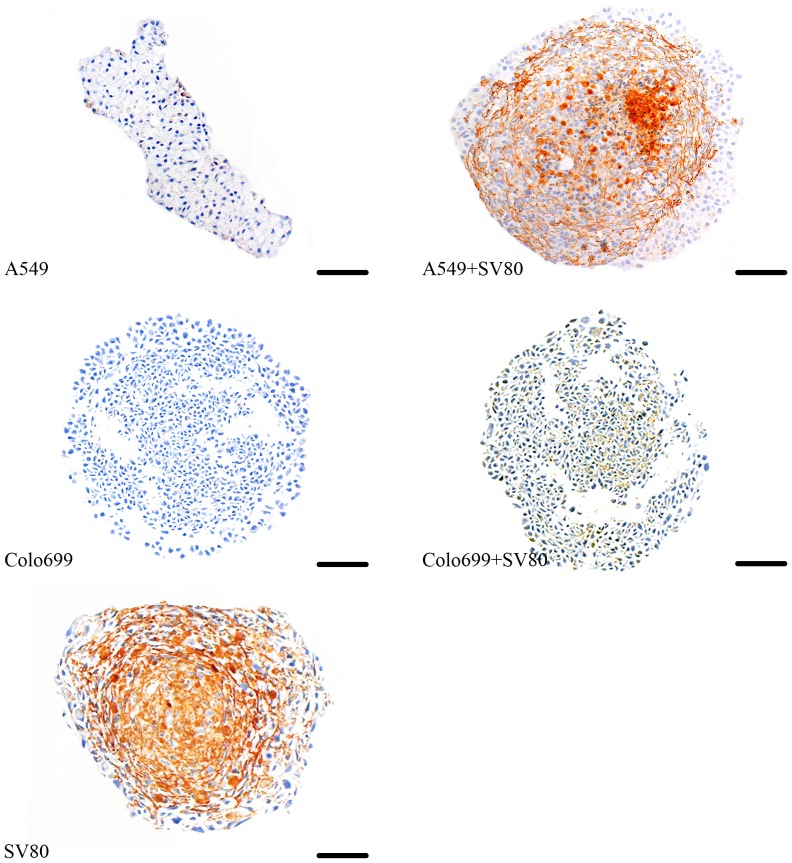
Extracellular matrix expression pattern. Fibronectin was displayed in microtissues after ten days. (Bar in A549/SV80 monocultures: 50 μm, bar in all other microtissues: 100 μm); Secretion of fibronectin could be observed in microtissues containing fibroblasts, whereas in all tumour cell monocultures no fibronectin secretion could be detected.

As all co-cultures showed a more homogenous surface with a tight architecture, we decided to examine whether this depends on ECM production of the cells. In figure 3 we demonstrate that the SV80 cells are significantly producing fibronectin when cultivated on their own, whereas both tumour cell monocultures were completely negative for fibronectin. In Colo699 co-cultures ECM was evenly distributed throughout the whole microtissue. However, in A549 co-cultures fibronectin was mainly secreted by cells that built up the ring like structure around the inner core of the microtissue.

### Growth curve and stability of tumour microtissues

**Figure 4:**
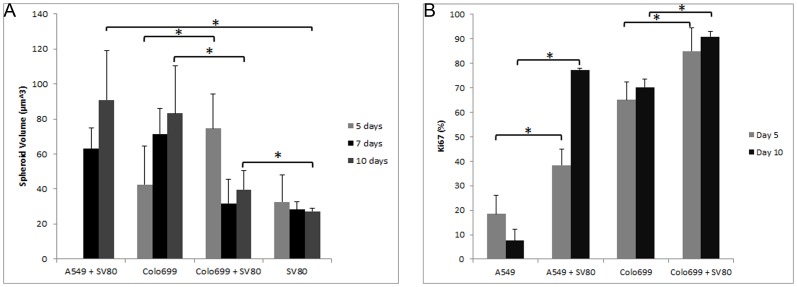
Growth curve and Cell proliferation/activation pattern. **A**: Microtissue volumes were calculated by the formula V = 4/3*Π*r3, where r = ½*√ d1*d2. Mean volumes are displayed in μm3 with their corresponding standard deviations. **B**: Mean and standard deviation of Ki67 positivity was calculated by counting Ki67 positive and negative cell nuclei on slices of three different spheroids representatively. Thereafter, the percentage of positive cell nuclei to whole cell cumber was calculated. Co-cultivation of both tumour cell lines with a fibroblast cell line led to a significant upregulation (p<0,05) of Ki67 expression in all microtissues.

Three microtissues of mono- and co-cultures were evaluated by light microscope and after five, seven and ten days microtissue volumes were calculated (figure 4A).

It was not possible to calculate spheroid volumes of A549 microtissues because in monocultures these cells did not form round or ellipsoid shaped spheroids. The same problem evolved with the A549 co-cultures that only started to form round measurable spheroids after five days. Therefore, the first A549 co-culture volume calculation was done on day seven, where a volume of 63 μm^3^ was measured. During the course of three days the volume of this co-culture increased to 81 μm^3^.

Colo699 monocultures displayed a continuous increase in volume from 43 μm^3^ to f 83 μm^3^. In contrast, Colo699 co-cultures showed a volume of 74 μm^3^ after five days with a decrease in volume to 39 μm^3^.

In SV80 monocultures a steady volume of 33 μm^3^ after five days to 27 μm^3^ after ten days could be observed.

Finally, microtissue volumes were compared to one another with the student's t-test. After ten days a significant increase of volume could be measured in A549 co-cultures and Colo699 monocultures when compared to either SV80 monocultures or Colo699 co-cultures. On the other hand, after five days the volume of Colo699 co-cultures had been significantly higher compared to Colo699 and SV80 monocultures (figure 4A).

**Figure 5:**
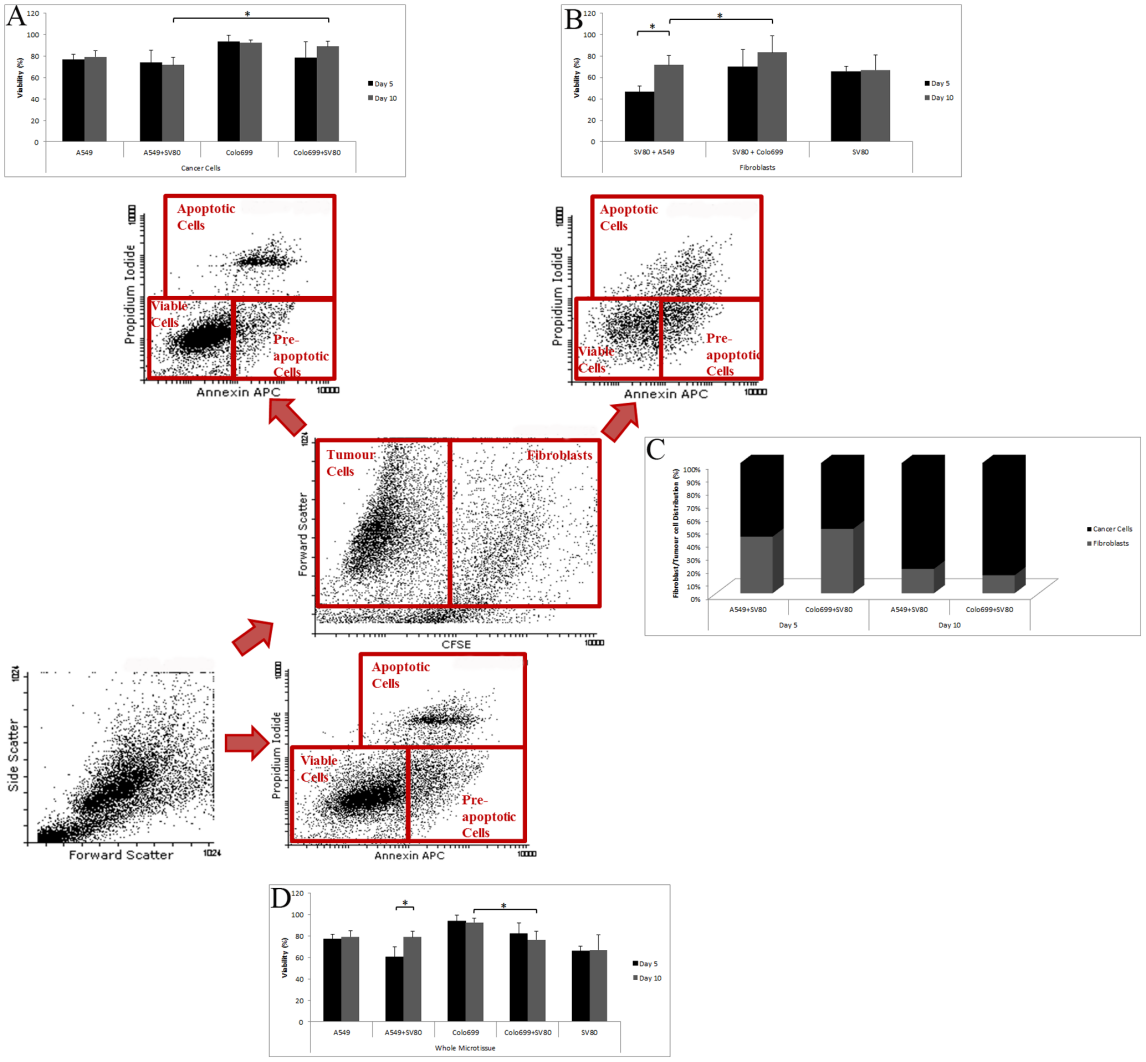
Viability of microtissues was measured with an Annexin V and Propidium Iodide protocol for flow cytometry. Fibroblasts were incubated with CFSE before initially seeding them together with tumour cells in the hanging drops. Apoptosis was measured either after five or ten days. Each bar represents the mean cell viability of 24 spheroids and their corresponding standard deviation that were measured in two different runs (Significant differences are marked with *, p<0,05). Dot blots represent the FACS analyses of one run containing A549 cells in co-culture with SV80 fibroblasts after ten days of cultivation. A: Specific viability of cancer cells in mono-and co-cultures are displayed. B: viability of solitary fibroblasts in mono-and co-cultures are displayed. C: The distribution between fibroblasts and tumour cells after five and ten days is displayed. D: Whole microtissue viability is shown after five and ten days.

To investigate cell viability, an Annexin V APC and Propidium Iodide (PI) FACS assay was established. SV80 cells were incubated with CFSE before seeding, thus viability of cancer cells and fibroblasts could be measured independently by discrimination in the FACS analyses. Microtissues were harvested, digested with Accumax and incubated with PI and Annexin V APC. Analyses were performed on a BD FACS Calibur system. Each bar represents the mean cell viability of 24 different microtissues measured in three independent runs. For statistical evaluation the student's t-test was used (figure 5).

A549 monocultures displayed 77% of viable cells after five days with a minimal increase in viability after ten days to 79%. Co-cultures with fibroblasts showed a significant increase in viability from 60% after five days to 79% after ten days. Colo699 cells displayed 93% of viable cells after five days with no decrease in viability to 92% after ten days. In co-cultures 82% of viable cells persisted after five days and 76% after ten days. When viability of SV80 cells was analysed, a minor increase from 65% after five days to 67% after ten days was shown. However, a significant decrease in viability from Colo699 monocultures to Colo699 co-cultures could be observed (figure 5D).

As a next step, viability of the two different fractions, tumour cells and fibroblasts were analysed by discriminating them by flow cytometry. A549 cancer cells in the co-cultures remained viable throughout the incubation with a viability of 74% after five days and 71% after ten days. In Colo699 co-cultures an increase in viability could be observed from 78% after five to 89% after ten days, showing a significantly better viability when compared to A549 co-cultured cancer cells after ten days (figure 5A).

A similar observation was made regarding the viability of fibroblasts in the microtissues. When co-cultivated together with the A549 cancer cells, SV80 cells displayed a significant upregulation in viability from 46% after five days to 71% after ten days. In the Colo699 co-cultures the viability also increased but not significantly varying between 70% after five to 83% after ten days. Simultaneously, a significant better viability for fibroblasts when co-cultivated together with Colo699 cells after five days could be displayed when compared to A540 co-cultures (figure 5B).

Furthermore, the ratio between fibroblasts and tumour cells in co-cultures was analysed after five and ten days. When co-cultures were grown in the hanging drop, cancer cells and fibroblasts were always seeded in a ratio of 1∶2 (2500 cancer cells/ 5000 fibroblasts). After five days in both co-cultures a nearly even distribution between fibroblasts and cancer cells could be observed, varying from 43% fibroblasts to 57% tumour cells in the A549 co-cultures and 49% to 51% in the Colo699 co-cultures. However, after ten days significantly more tumour cells than fibroblasts in both co-cultures could be displayed. In A549 co-cultures, 19% and in Colo699 co-cultures 14% of all cells were identified as fibroblasts (figure 5C).

### Cell organization and protein expression pattern

**Figure 6:**
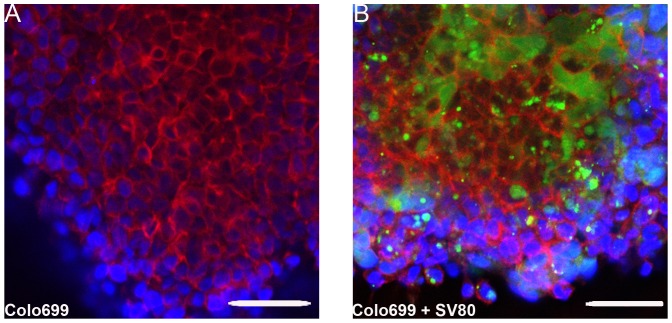
Fluorescence microscope images of Colo699 either in mono- or co-culture. (A) Colo699 monocultures after ten days; (B) Colo699 co-cultures after ten days; DAPI (blue) was used for staining cell nuclei, Phalloidin Atto 633 (red) for displaying F-actin and CFSE (green) as a cytoplasmic stain for fibroblasts (Bar in A/B: 50 μm). Fibroblasts could still be detected in microtissues after five days of co-cultivation with Colo699.

**Figure 7:**
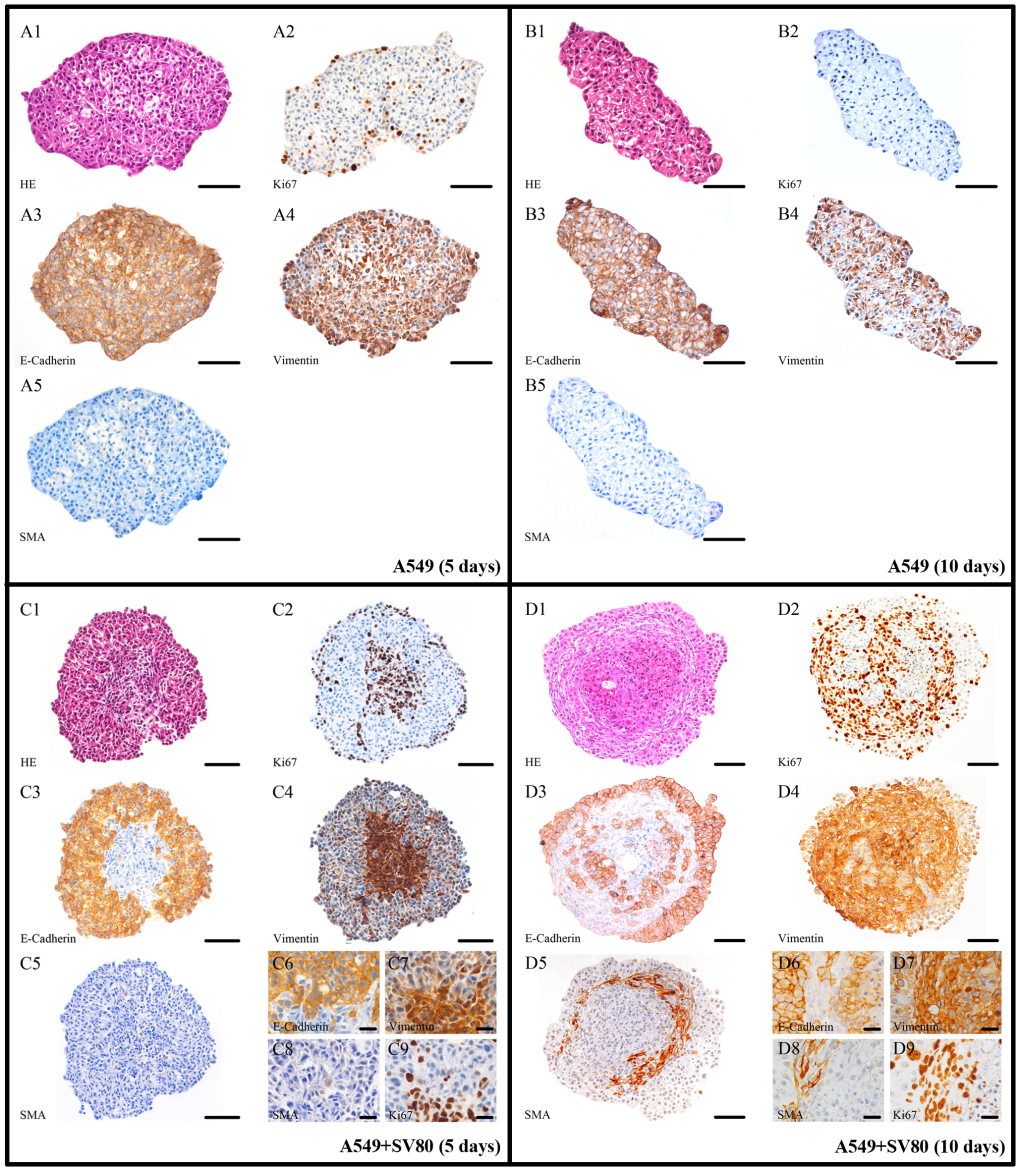
A549 microtissue protein expression pattern. IHC slices of A549 in mono- and co-cultures after five and ten days (Bar in A-D: 100 μm, bar in Insert: 25 μm); an increase in vimentin and a simultaneous decrease in E-Cadherin is displayed in co-cultures. Also an upregulation of Ki67 expression in co-cultures was detected.

**Figure 8:**
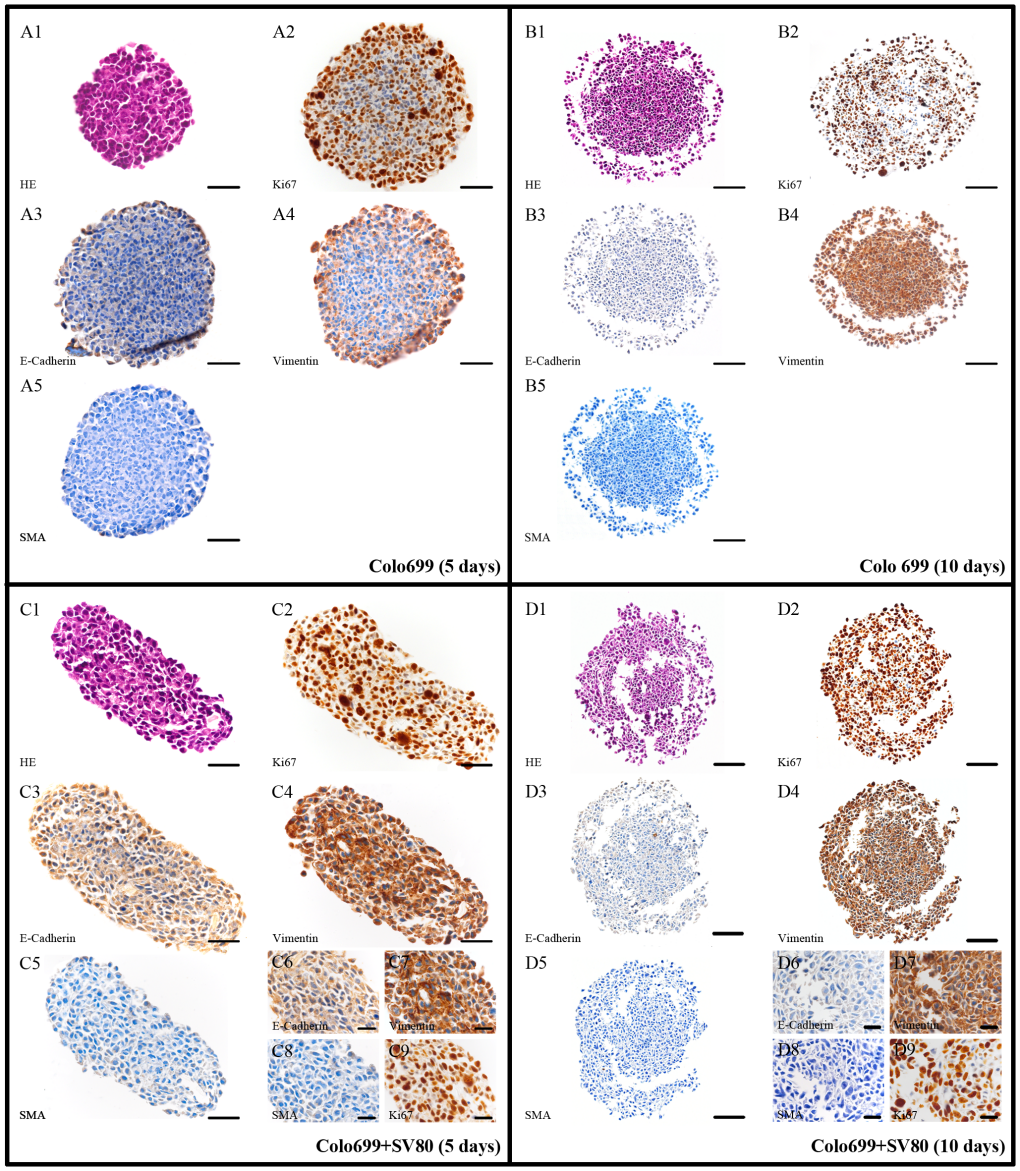
Colo699 microtissue protein expression pattern. IHC slices of Colo699 in mono- and co-cultures after five and ten days (Bar in A/C: 50 μm, bar in B/D: 100 μm, bar in Insert: 25 μm); Compared to monocultures Colo699 co-cultures formed much smaller spheroids. However, positive staining reactions for E-Cadherin and vimentin are comparable to the monocultures in the period of ten days. after ten days because no difference in the staining pattern between five and ten days was detected. Microtissues were completely positive for vimentin and Ki67, whereas no E-Cadherin and α-SMA expression was observed.

**Figure 9:**
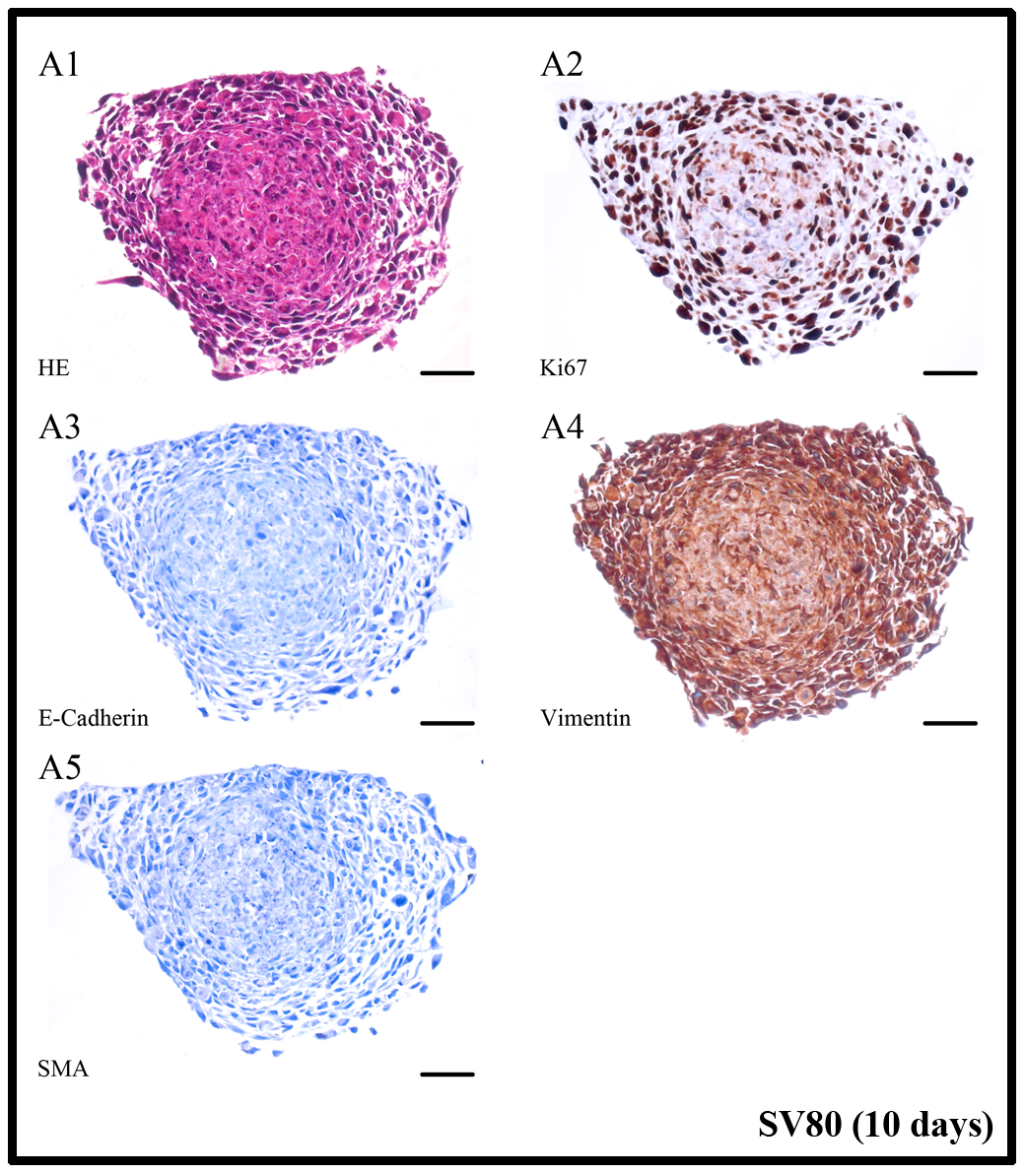
SV80 microtissue protein expression pattern. IHC slices of SV80 in monocultures after ten days (Bar in A: 100 μm); SV80 microtissue staining is only shown representatively after ten days because no difference in the staining pattern between five and ten days was detected. Microtissues were completely positive for vimentin and Ki67, whereas no E-Cadherin and α-SMA expression was observed.

After incubating A549 and Colo699 cells alone or in co-culture with fibroblasts for five and ten days, immunohistochemical analyses were performed (figure 5–7/table 2). Vimentin was chosen as a protein indicating a shift to a mesenchymal phenotype in epithelial cells. α-SMA expression indicates an even more mesenchymal phenotype of fibroblasts and reflects a mesenchymal to mesenchymal transition. E-Cadherin, an epithelial cell adhesion protein was chosen as a marker for adherent cells and Ki67 as a cell proliferation and activation marker. Three different spheroids were analysed for each protein staining and consecutive slices of one spheroid are shown representatively here (figure 7–9).

**Table 2:** Summary of IHC protein expression pattern. +/- displays the presence/absence of the indicated protein.

Cell lines	A549		A549+SV80		Colo699		Colo699+SV80		SV80	
Days	5	10	5	10	5	10	5	10	5	10
**E-Cadherin**	+	+	+	+	+	−	+	−	−	−
**Vimentin**	+	+	+	+	+	+	+	+	+	+
**α-SMA**	−	−	−	+	−	−	−	−	−	−
**Ki-67**	+	−	+	+	+	+	+	+	+	+
**Fibronectin**	−	−	+	+	−	−	+	+	+	+
**Cytokeratin 7**	+	+	+	+	−	−	−	−	−	−

In A549 cells an upregulation of vimentin was demonstrated by being more expressed in co-cultures compared to monoculture. In contrast, E-Cadherin expression progressively decreased during the cultivation period of ten days. Compared to monoculture, this downregulation was significantly more expressed in co-cultures with fibroblasts. No up-regulation of Ki-67 could be detected in A549 cells when cultivated alone. In contrast, when co-cultivated together with fibroblasts, with fibroblasts, tumour cells significantly expressed Ki-67 especially in the outer parts of the spheroids and also partially in the spheroid cores (figure 6/table 2). On the other hand, SV-80 cells showed a strong expression pattern throughout the whole spheroid (figure 9/table 2).

In the inserts of figure 6 (C6–C9 and D6–D9) a small area is shown in a higher magnification. After ten days (figure 6 D6–D9) tumour cells can be found in the fibroblast ring discriminable by an expression of E-Cadherin. Especially in these tumour cells in addition to E-Cadherin, vimentin and Ki67 is highly expressed.

In Colo699 mono- and co-cultures vimentin was already expressed after five days with a slight upregulation in the co-cultured tumour cells. After ten days mono- and co-cultures similarly showed a high expression of this mesenchymal protein. Both, mono- and co-cultures demonstrated no significant expression of E-Cadherin either after five or after ten days. Furthermore, Ki-67 is expressed both in mono- and co-cultures after five and ten days. A significant upregulation of this protein was detected in co-cultures after five days throughout the whole spheroid, whereas in monocultures only cells near the spheroid surface expressed this protein (figure 7/table 2).

**Figure 10:**
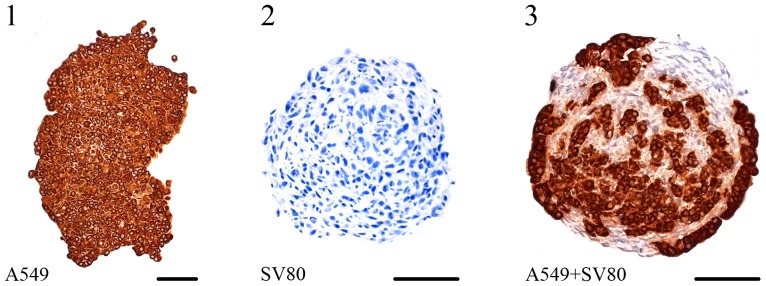
Cytokeratin 7 expression pattern. IHC staining of A549, SV80 monocultures and A549 co-cultures after ten days (Bar in 1–3: 100 μm). Cytokeratin 7 staining could only be observed in cancer cells but not in SV80 cells.

A limitation of the immunohistochemically stained slides of Colo699 mono- and co-cultures was the fact that no differentiation between fibroblasts and tumour cells could be made. In contrast, in A549 co-cultures fibroblasts could clearly be discriminated from cancer cells due to their different morphology and spheroid location as well as the strong expression of vimentin, α-SMA and the negative expression for cytokeratin 7 (figure 6 and 10). Therefore, we used an immunofluorescence microscope screening whether CFSE labeled fibroblasts could be detected in Colo699 co-cultures. After five days of incubation, CFSE labeled fibroblasts can still be observed and are represented predominantly in the spheroid core (figure 6).

While A549 and Colo699 cells did not express α-SMA, SV-80 fibroblast cells showed an increase in the expression of α-SMA after ten days in co-cultivation with A549 cells (figure 7). Cytokeratin 7 staining was then used to further distinguish between cancer cells and α-SMA expressing fibroblasts (figure 10). Cytokeratin 7 positive cancer cells cannot only be found settled in the centre of the microtissue but also scattered in the fibroblast ring and in the periphery of the microtissue. IHC expression pattern is only shown after ten days for the fibroblast monocultures because no difference could be observed between five and ten days. In monocultures SV80 microtissues remained negative for E-Cadherin and α-SMA expression while all cells expressed Ki67 and vimentin (figure 9/table 2).

### Cell proliferation and activation pattern

As all co-cultures showed an increase in Ki67 expression, we determined the exact percentage of Ki67 positive to negative cells by counting Ki67 positive and negative cell nuclei on slides of three different spheroids (figure 4B).

A549 monocultures showed a decrease in Ki67 positive cells from 18% after five days to 7%

after ten days. In contrast, in A549 co-cultures 38% of cells were positive after five days with a significant increase to 77% after ten days. A minor increase of Ki67 positive cells from 65% to 70% could be observed in Colo699 monocultures. In co-cultures of Colo699 microtissues an increase of Ki67 positivity was detected from 84% to 90% after ten days, despite the decrease in viability and volume after ten days (figure 4A/5D). A significant upregulation (p<0,05) of Ki67 expression was found in all co-cultures when compared to monocultures.

## Discussion

We describe a novel co-culture method using two different non-small cell lung cancer (NSCLC) cell lines together with a lung fibroblast cell line in the hanging drop technology for the investigation of tumour-stroma interactions. NSCLC cells were chosen because several targeted therapies have recently failed to show major benefit in clinical trials despite promising in vitro data [6]. Therefore, the challenge for developing novel targeted therapies is the establishment of in vitro models that better reflect in vivo conditions. Subgroups of patients could then be identified and benefit most from the respective drug. 3D cell co-culture techniques using cancer cells in combination with stroma cells represent a promising approach to achieve this goal.

So far, standard 3D systems use cell spheroid aggregates and scaffold culture systems. These systems allow 3D cell growth by extracellular mechanical support and thereby support cell adhesion and aggregation. Other 3D-systems use liquid overlay technologies, fibre meshwork made of biocompatible polymers, solid or porous beads and extracellular matrixes or substitutes thereof and thus require the addition of supplements for spheroid growth [11]–[14].

Existing 3D tumour cell culture models mainly consist of tumour cells in monocultures whereas when co-cultured together with fibroblasts these systems always require either additional growth factors or exogenously produced ECM and have limited cultivation times. Therefore, with conventional 3D cultures neither long term drug effects nor tumour-stroma interactions can be studied without influence of non-physiological matrices.

To overcome these limitations the hanging drop method was developed further to allow the generation of multicellular spheroids without growth factor supplementation and addition of non-physiological matrices on the one hand and perform regular medium exchanges on the other [24].

We demonstrate that both monocultures show an improved viability when cultivated alone. Together with fibroblasts in both co-cultures a decrease in viability during the course of the co-culturing could be observed. The reduced viability can be explained by the generation of more compact spheroids in the co-culture setting by fibroblast related production of fibronectin. This leads to an enhancement of cancer cell adherence, increased hypoxic conditions and limited nutrition diffusion, thereby inducing necrosis, apoptosis in tumour cells and restricting spheroid growth [26], [27]. In addition, especially in co-cultures of A549 cells an upregulation of vimentin could be observed suggesting an epithelial to mesenchymal transition (EMT) in tumour cells as previously reported [28].

Despite the fact that both cancer cell lines are adenocarcinoma derived, protein pattern differs. In the description of the company (DSMZ) where all cell lines were purchased from, it is indicated that the Colo699 cells are more mesenchymal derived cells, expressing vimentin and no cytokeratin. A549 cells on the other hand, apart from vimentin also express cytokeratin (7,8,18,19). Therefore, adhesion molecules expressing A549 cells benefit most when co-cultured together with fibroblasts secreting extracellular matrix. These co-cultures keep a heterogeneous microtissue architecture, where tumour cells can still be discriminated by the cell adhesion molecule E-cadherin.

On the other hand, the co-incubation of cancer cells and SV80 fibroblasts led to a significantly increased expression of Ki67. This protein is expressed in all cell cycle phases but not in G0, thereby indicating not only proliferation but also enhanced activity of Colo699 co-cultures [29]. As Colo699 co-cultures showed a reduced viability and a significantly lower spheroid volume after ten days of incubation, Ki67 indicates not so much proliferation but high metabolic activity of tumour cells. Not only in co-cultures an increase in Ki67 expression during cultivation could be observed but also SV80 monocultures displayed a high expression of this protein. However, even in fibroblast monocultures no increase in volume could be observed. Despite that the SV40 immortalization in cells has been shown to induce Ki67, the positivity of these cells is therefore not derived from proliferation but activation [30].

Because of the determination of the ratio between fibroblasts and tumour cells by FACS analyses it can be excluded that all Ki67 positive cells in both co-cultures after ten days are tumour cells. Transformed fibroblasts may not resemble in vivo stromal cells in patients. However, a novel co-culture system was developed with the goal of establishing a well-defined and repeatable cell culture model. Therefore, immortalized fibroblasts were used to lower the risk of growing microtissues for several weeks with limited comparability.

α-SMA expression is a marker that indicates mesenchymal to mesenchymal transition in fibroblasts. These transformed fibroblasts are called cancer associated fibroblasts and are known to support survival of cancer cells by secretion of growth hormones and cytokines [31]–[33]. No data can be found where α-SMA expression and Ki67 expression are correlated. In our system α-SMA expression could only be detected in A549 co-cultures. Therefore, cytokeratin 7 staining, a protein only expressed by cancer cells, was performed to prove that only fibroblasts generated α-SMA in our microtissues. The expression pattern of cytokeratin 7 positive cancer cells in A549 co-cultures indicates that all α-SMA expressing cells are at the same time cytokeratin 7 negative. Thus it can be concluded that only fibroblasts express this cancer associated fibroblast marker. Besides the transition of fibroblasts, these microtissues also displayed the highest increase of Ki67 positive cells after ten days of co-cultivation.

Beside a change in Ki67/α-SMA expression in co-cultures also an increase of viability in fibroblasts when co-cultivated together with cancer cells could be observed. In both co-cultures fibroblast viability increased during cultivation and after ten days in both co-cultures a higher percentage of fibroblasts still remained viable when compared to SV80 monocultures.

Our data suggest that in our 3D cell culture model the interaction of tumour cells and fibroblasts not only results in activation of tumour cells but also to an increase of viability and induction of myofibroblast proteins of stroma cells.

## Conclusion

We demonstrate that our hanging drops technology is a promising tool for the generation of tumour spheroid co-cultures. Our method allows investigations of tumour-stroma interactions with a reflection of the in-vivo condition. Therefore, we will use this model in comparative drug testing in comparison to 2D cultures to obtain not only deeper insights in tumour pathobiology but also in drug efficacy.
